# Effect of Volatile Organic Compounds on Pulmonary Functions Among Paint Industry Workers of Unorganized Sectors

**DOI:** 10.7759/cureus.58951

**Published:** 2024-04-24

**Authors:** Lavanya Sekhar, Teena Lal, Vidhya Venugopal, Santhanam R, Priscilla Johnson

**Affiliations:** 1 Physiology, Sri Ramachandra Medical College & Research Institute, SRIHER (DU), Chennai, IND; 2 Physiology, Sri Lalithambigai Medical College Hospital, Chennai, IND; 3 Environmental Health Engineering, Sri Ramachandra Institute of Higher Education and Research, SRIHER (DU), Chennai, IND; 4 Neuro Surgery, Sree Balaji Medical College & Research Institute, Chennai, IND; 5 Department of Physiology, Sri Ramachandra Medical College & Research Institute, SRIHER (DU), Chennai, IND

**Keywords:** unorganized sectors, paint manufacturing workers, volatile organic compounds, respiratory symptoms, spirometry, peak expiratory flow rate, paint industry workers, construction painters

## Abstract

Background

Paint industry workers are constantly exposed to paints and organic solvents that contain a substantial quantity of volatile organic compounds (VOCs). Exposure to VOC emissions could result in pulmonary, neurobehavioral, and hematological consequences. Limited studies have been undertaken in India to assess the health consequences of VOCs among paint industry workers in unorganized sectors.

Aim

To assess the effects of VOCs on pulmonary function in paint industry workers of unorganized sectors.

Methodology

A hundred and twenty full-time male construction painters and small-scale paint manufacturing workers aged 25-60 were assessed for respiratory symptoms using a questionnaire, and pulmonary functions using Wright's Peak Expiratory Flow Meter (PEFR). Participants were randomly selected for VOC assessment and the cumulative solvent exposure index was calculated. A pulmonary function test (PFT) was performed on a subset of construction painters (n=30) using a Koko spirometer.

Results

The concentration of VOCs such as benzene, ethylbenzene, toluene, and xylene (BETX) and dichloromethane levels exceeded American Conference of Governmental Industrial Hygienists (ACGIH) threshold limit values (TLVs) among the paint manufacturing workers. About 52% of paint workers reported respiratory symptoms. Around 22% of the participants showed reduced pulmonary function (PEFR<400 L/min). There was a significant weak negative correlation between PEFR and work experience (r = -0.2, p=0.03). PFT parameters among a subset of construction painters revealed a significant moderate negative correlation with work experience [forced expiratory volume at the onset of the first second (FEV1) (r = -0.6, p=0.001) and forced vital capacity (FVC) (r = -0.53, p=0.005)] and cumulative VOC exposure index [FEV1 (r = -0.53, p = 0.004) and FVC (r = -0.5, p = 0.008)].

Conclusion

The concentration of VOCs was higher among paint industry workers of unorganized sectors and they reported respiratory symptoms and diminished pulmonary function. To reduce morbidity, it is critical to enhance awareness about occupational safety and services in these unorganized sectors.

## Introduction

Volatile organic compounds (VOCs) are an array of organic compounds that exhibit high vapor pressure even at room temperature. This causes the constituent liquids or solids to readily evaporate or sublimate a significant number of molecules into the ambient air. VOCs possess enhanced mobility and resistance to degradation, enabling them to be conveyed over extended distances in the environment, in addition to their volatility [1].

Many household products, including paints, varnishes, cleaning and disinfecting products, cosmetics, etc., constitute organic solvents and release VOCs. All of these products emit organic compounds both while being used and stored [2]. Paints are manufactured utilizing an extensive variety of volatile organic compounds, which consist of over 30% of its constituents as they enable binding and quick drying [3] and occupations related to paints such as painters, paint industry workers are constantly being exposed to these compounds during a variety of activities such as spraying, rolling, mixing, drying and cleaning of paints [4, 5].

Various epidemiological studies conducted globally have documented respiratory, neurobehavioral, and hematological effects of exposure to VOCs present in paints and organic solvents [6-10]. However, the majority of these studies have focused on organized sectors such as the automobile industry, shipyard painters, and large-scale paint workers. Limited research has been conducted on the health impacts of VOCs among paint industry workers in unorganized sectors, such as construction and small-scale paint manufacturing industries, where there is a lack of knowledge about the harmful effects of VOCs and their health consequences. Further, there is minimal awareness about the availability and use of personal protective equipment (PPE) by both employers and employees in these sectors. Hence, this descriptive cross-sectional study aims to assess the effect of VOCs on the pulmonary functions of unorganized paint industry workers.

## Materials and methods

Study design

The objective of this cross-sectional study is to evaluate the prevalence of respiratory symptoms and pulmonary functions among paint industry workers and to assess the effect of VOC on pulmonary functions in paint industry workers exposed to paints and organic solvents in the unorganized sector.

Ethical consideration

The current study was carried out in adherence to the Helsinki Declaration and the ICMR's Code of Ethics for Biomedical and Health Research, with approval from the Institutional Ethics Committee (Registration No. IEC-NI/21/FEB/77-33). 

Inclusion and exclusion criteria

Male construction painters and paint manufacturing workers aged 25 to 60 were included in the study. Further, workers who had recently undergone ocular or thoracoabdominal procedures, congenital or acquired cardiorespiratory disorders, tuberculosis, recent respiratory infections, or any manifestations of COVID-19 were excluded.

Sample size determination

The sample size was calculated using the formula n = Z21-α/2 PQ/L2, where Z = 1.96 [95% confidence interval (CI)] and P is the prevalence (P) of respiratory symptoms (wheezing: 7.5%) among spray painters exposed to volatile organic compounds and paint solvents in Nigeria [10], Q=1-P, and with a tolerable level of error (L) of 5% and the appropriate sample size (n=107) was derived.

Selection of study participants

**Figure 1:**
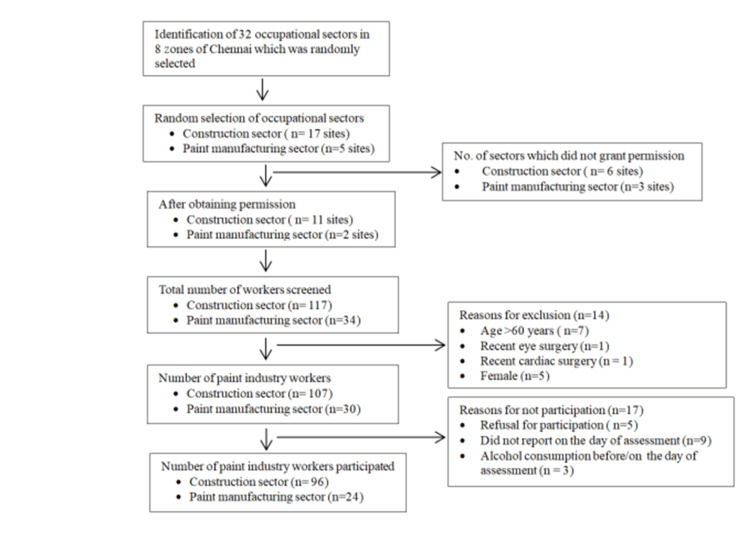
Flowchart of subject recruitment and selection

Eight out of fifteen different zones of Chennai and its suburbs were randomly selected and 32 sites were identified from these selected zones by contacting the painter's associations and the local hardware shops in these zones. Using a random number generator, 22 small-scale paint industries (seventeen small-scale construction sites and five paint manufacturing industries) were selected. Eleven construction sites and two small-scale paint industries granted permission to conduct the study and a total of 151 paint industry workers (117 construction painters and 34 small-scale paint industry workers) were screened for eligibility. Following screening 120 male paint industry workers (full-time) aged 25 to 60 years (n=96 from construction painters, n=24 paint manufacturing workers) fulfilled the predetermined inclusion criteria, accepted to participate, and were subsequently enrolled in the study (Figure 1).

Written informed consent was obtained from all study participants who met the inclusion criteria in their local language. A standardized questionnaire was utilized to obtain general information including age, literacy status, socioeconomic status, smoking status, alcoholic status, and medical history. Additionally, a comprehensive history of VOC exposure, including information such as the number of years of work experience, the predominant type of paint used, and the method of application was obtained. Furthermore, information concerning the perception and utilization of personal protective equipment (PPE) was also obtained from the study participants.

VOC exposure assessment

Assessment of volatile organic compound (VOC) exposure was conducted in the paint industries and construction sites by active sampling method and a portable personal air sampler. Thirty randomly selected study participants were subjected to direct personal VOC exposure monitoring while performing various kinds of tasks, including enamel brushing (n=10), emulsion brushing (n=7), paint scraping/stripping (n=7) from the construction industry, and paint stirring (n=3) and paint filling (n=3) from the paint industry. Active sampling was performed using a calibrated, battery-powered sampling pump connected to a solid sorbent tube via flexible cabling [11], Active sampling involved the withdrawal of a predetermined volume of air through an absorbent medium composed of carboxean and tenax and the contaminants were accumulated via adsorption. VOCs were subsequently extracted from the samples and were then analyzed using a gas chromatography-mass spectrometry (GCMS) agilent 7890B/5977B GCMSD-54 VOC analyzer. The concentrations of VOCs present in paints such as benzene, xylene, ethyl benzene, toluene (BETX), and dichloromethane were measured in parts per million (ppm).

The cumulative exposure index (CEI) was computed for each participant in ppm-years utilizing the solvent exposure index developed by Lee et al. [12]. This index is derived from the product of the duration of exposure and the mixed solvent exposure index (En).

CEI=En (ppm) x exposure duration (years),

where the mixed organic solvent exposure index (En) was calculated by dividing the sum of each current VOC level (Ci) by the threshold limit value (TLVi) of each solvent as established by the American Conference of Governmental Industrial Hygienists (ACGIH) (En =  ​​∑Ci/TLVi).

Health assessment

The health assessment was performed on an exposure-free day lasting at least 24 hours, and the study participants were instructed not to consume any alcoholic beverages. Using a respiratory symptoms questionnaire and Wright's Peak Flow Meter, respiratory symptoms including wheezing, nasal/throat irritation, and cough history, as well as pulmonary functions including peak expiratory flow rate (PEFR), were evaluated for all 120 study participants. Additionally, lung function tests, including forced expiratory volume at the onset of the first second (FEV1), forced vital capacity (FVC), and FEV1/FVC, were evaluated following American Thoracic Society (ATS) and European Respiratory Society (ERS) guidelines [13] on a subset of 30 construction painters who were predominantly exposed to solvent (enamel) paints using KOKO spirometers.

Data analysis

The statistical analysis was conducted using the R software version 4.2.1 (R Foundation for Statistical Computing, Vienna, Austria). Descriptive parameters are represented in the form of frequency and percentage for categorical variables, while mean and standard deviation, median, and range are utilized to represent continuous variables. Statistical significance was evaluated using chi-square and independent t-tests; a p-value less than 0.05 was considered to indicate statistical significance. Pearson's correlation was utilized to calculate the correlation between the continuous variables.

## Results

This cross-sectional study included 120 male participants from unorganized industrial sectors which comprised 96 construction painters and 24 small-scale paint manufacturing workers. The mean age of the study participants was 36.95±10.57 years and their average body mass index (BMI) was 22.46±4.7 Kg/m2. The mean years of work experience of the study participants was 12.52±7.7 years, with 15 individuals (12.5%) possessing more than 20 years of work experience. Almost 72% of painters were alcoholics, whereas 32% were active smokers, with an average pack-year of 1.69±3.47. About 73% of the participants in the study possessed knowledge about the importance of donning personal protective equipment. Specifically, 11% of the study participants affiliated with the construction industry and 62% from the paint manufacturing sector indicated that surgical masks were readily available at their respective work sites. The majority of construction painters stated that they rarely utilized cloth masks (50%) and surgical masks (15%), only 6% were observed to be proficient users. A summary of the demographic characteristics of the study participants is provided in Table 1.

**Table 1:** Descriptive characteristics (n=120) BMI: body mass index, PPE: personal protective equipment.

Variables		n(%)
Age (years)	≤35	61(51)
	>35	59 (49)
BMI (Kg/m^2^)	<18	19(16)
	18 - 25	76 (63)
	>25	25 (21)
Work Experience (years)	≤10	65(54)
	>10	55 (46)
Occupation	Construction painters	96 (80)
	Paint manufacturing workers	24 (20)
Smoker	Yes	39(32)
	No	81 (68)
Alcohol consumption	Yes	86 (72)
	No	34 (28)
Socio economic status	Upper Middle	11 (9)
	Lower	109(91)
Locality	Rural	38 (32)
	Urban	82 (68)
Housing	Kucha	21 (18)
	Pucca	99 (82)
PPE awareness	Yes	88(73)
	No	32(27)
PPE usage	Yes	7(6)
	No	113(94)

While assessing the primary paint types used by the majority of construction painters, with a usage rate of more than 90%, it was found that approximately 57% of construction site painters used emulsion paints (water-based), 22% used enamel paints (solvent-based), and nearly 21% used powder/putty. Almost 82% of the construction painters reported performing paint scraping and stripping of the old buildings before painting. When investigating the predominant techniques utilized during the painting process, it was found that over 70% of painters utilized rolling/brushing with enamel/emulsion paints, and less than nine percent utilized enamel and emulsion spraying. Moreover, within the paint manufacturing sectors, approximately 37% of employees were engaged in activities related to the preparation of raw materials, such as weighing, drying, and dissolving. Paint mixing, grinding, and filtration constituted the work of 42% of the workforce, while paint filling and packaging occupied the remaining 10%. However, nearly 11% of the employees in the paint industry reported that they were involved in all the activities depending on the requirements of the workforce. Both sectors of the paint manufacturing industry manufactured oil emulsion paints containing solvents and no formal practice of job rotation was followed in any of these industries.

Airborne concentrations of major VOCs isolated from paints are shown in Table 2. It was found that paint manufacturing workers are exposed to higher concentrations of all VOCs than construction painters, and the median levels of ethylbenzene, toluene, O-xylene, and dichloromethane exceeded ACGIH threshold values (TLVs). Additionally, it was observed that the median levels of VOCs in construction painters were lower than the ACGIH TLVs. Nevertheless, certain samples of construction painters engaged in enamel brushing and paint scraping in a confined environment, where concentrations of ethylbenzene, toluene, and o-xylene ranged higher. In the construction industry, the mixed VOC exposure index (En) for dichloromethane and BETX was 0.95 ppm, while in the paint manufacturing industry, it was 3.19 ppm. The study participants exhibited a cumulative exposure index (CEI) ranging from 6.68 to 79.81 ppm-years, with a median value of 16.85 ppm-years.

**Table 2:** Airborne concentration of major VOCs in paints ppm: parts per million, ACGIH-TLV: American Conference of Governmental and Industrial Hygienists - threshold limit values

Volatile Organic Compounds	Construction Industry		Paint Manufacturing Industry		
	Median(ppm)	Range(ppm)	Median(ppm)	Range(ppm)	ACGIH -TLV (ppm)
Benzene	0.01	0.01-12.7	0.35	0.01-0.7	0.5
Ethyl benzene	1.63	0.01-521.9	153.9	2.35-305.6	20
Toluene	5.6	0.01-1168.3	229.6	93.5-365.6	20
o-Xylene	1.14	0.1-227.1	105.75	1.4-210.1	100
m-xylene	0.13	0.03-2.6	1.17	0.01-2.33	
p-xylene	0.6	0.04-0.93	58.5	0.5-116.5	
DiChloromethane	0.86	0.31-1.3	142.7	3.5-281.9	50

**Figure 2:**
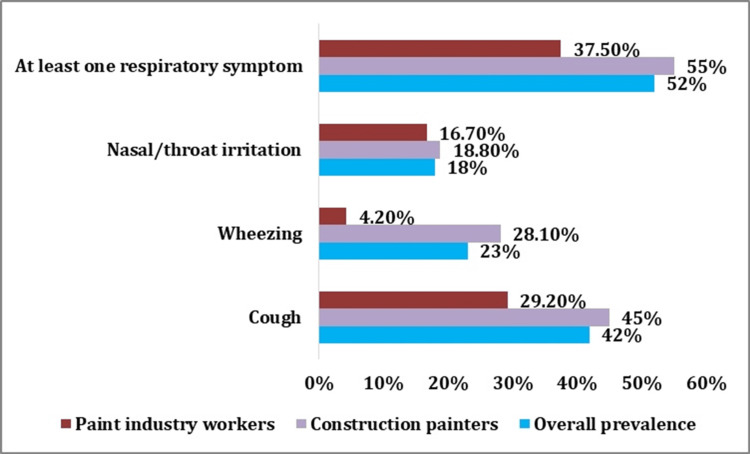
Prevalence of respiratory symptoms among paint industry workers

The prevalence of respiratory symptoms among unorganized paint industry workers (Figure 2) was found to be 52%. Individual respiratory symptoms such as cough, wheezing, and nasal/throat irritation were 42%, 23%, and 18%, respectively. While assessing the prevalence in each sector, it was found that 55% of construction painters and 37.5% of paint manufacturing workers experienced at least one of the respiratory symptoms. In addition, specific symptoms such as cough (45% vs 29.2%), wheezing (28.1% vs 4.2%), and nasal/throat irritation (18.8 vs 16.7%) were shown to be more prevalent in construction painters than in the paint industry workers.

In the current study, 22% of participants had reduced pulmonary functions (PEFR<400 L/min). While assessing the association between the PEFR and covariates (Table 3), it was found that there was a nonsignificant reduction in PEFR with increasing years of work experience. Further, age is a major factor that determines lung functions, and research participants above the age of 35 had considerably lower PEFR than younger ones. Furthermore, participants from rural parts of the Chennai suburbs had much lower PEFR than urban residents. However, there was no significant association between other factors such as the industrial sector, smoking, alcohol intake, and pulmonary function (Table* *3). Furthermore, we found a weak negative correlation (r=-0.2, p=0.03) between work experience and PEFR and between VOC exposure index and PEFR (r=-0.2, p=0.06).

**Table 3:** Association between PEFR and Covariates among paint industry workers (n=120) BMI: body mass index, PEFR (L/min): Peak Expiratory Flow Rate represented as liters /min

Variables		PEFR (L/min) (Mean± SD)	p value
Age (years)	≤35	500.33±74.18	0.02
	>35	459.15±66.37	
BMI (Kg/m^2^)	<18	457.37±53.11	0.1
	18 - 25	489.74±80.23	
	>25	468±58.59	
Work Experience (years)	≤10	489.85±75.67	0.1
	>10	468.55±68.92	
Smoking status	Smoker	479.49±78.44	0.9
	Non-smoker	480.37±70.95	
Alcohol consumption	Yes	475.58±80.04	0.1
	No	491.47±51	
Socio economic status	Upper Middle	507.27±57.81	0.13
	Lower	477.34±74.17	
Locality	Rural	460±56.52	0.02
	Urban	489.39±78.24	
Housing	Kucha	474.29±57.41	0.6
	Pucca	481.31±76.25	
Occupation	Construction painters	481.56±75.75	0.6
	Paint manufacturing workers	474.17±62.69	

Spirometry assessment of 30 construction painters who are predominantly exposed (>90%) to solvent-based paints, showed that all lung function parameters, such as FEV1, FVC, and FEV1/FVC have significantly reduced (p<0.05) with an increase in years of work experience (Table 4). Furthermore, it was found that increasing age had a significant effect on PFT values (p<0.05). Moreover, there was a significant decrease in FEV1/FVC values among painters who were smokers (p=0.04).

**Table 4:** Association between pulmonary function test (PFT) and covariates among construction painters (n=30) BMI: body mass index, FEV1: Forced Expiratory Volume at the onset of the first second, FVC: Forced vital capacity, FEV1/FVC: ratio of FEV1 and FVC, *FEV1/FVC reported as proportions

Variables		FEV1		FVC		FEV1/FVC *	
		Mean ± SD	p value	Mean ± SD	p value	Mean ± SD	p value
Age (years)	≤35	3.13±0.39	0.01	3.64±0.57	0.002	0.86±0.06	0.09
	>35	2.3±0.47		2.79±0.62		0.82±0.06	
BMI (Kg/m^2^)	<18	2.85±0.64	0.24	3.24±0.5	0.2	0.87±0.07	0.35
	18 - 25	2.69±0.57		3.2±0.76		0.84±0.07	
	>25	2.23±0.6		2.74±0.65		0.81±0.04	
Work Experience (years)	≤10	2.32±0.49	<0.01	3.41±0.74	0.03	0.86±0.07	0.04
	>10	2.92±0.55		2.83±0.59		0.81±0.05	
Smoking status	Smoker	2.18±0.3	0.07	2.82±0.29	0.18	0.77±0.04	0.04
	Nonsmoker	2.68±0.6		3.16±0.75		0.85±0.06	
Alcohol consumption	Yes	2.48±0.58	0.1	2.95±0.76	0.1	0.84±0.06	0.9
	No	2.85±0.57		3.39±0.59		0.85±0.06	
Socio economic status	Upper Middle	3.02±0.48	0.08	3.61±0.58	0.08	0.84±0.02	0.8
	Lower	2.52±0.59		3±0.71		0.84±0.07	
Locality	Rural	2.6±0.53	0.8	3.09±0.76	0.8	0.85±0.07	0.46
	Urban	2.64±0.67		3.14±0.72		0.83±0.05	
Housing	Kucha	2.8±0.9	0.8	3.14±0.67	0.9	0.88±0.1	0.6
	Pucca	2.61±0.59		3.12±0.74		0.84±0.06	

**Figure 3:**
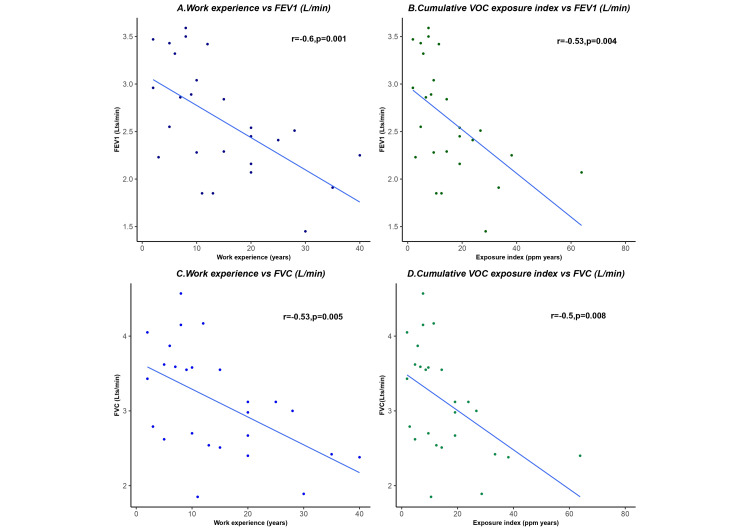
Effect of work experience and cumulative VOC exposure index on pulmonary function test (PFT) FEV1: Forced Expiratory Volume at the onset of the first second, FVC: Forced vital capacity, VOC: Volatile organic compounds

Figure 3 depicts the correlation between work experience and cumulative exposure index with PFT parameters among construction painters. The results showed a significant moderate negative correlation between work experience with FEV1 (r = -0.6, p = 0.001) and FVC (r = -0.53,p = 0.005) and between cumulative VOC exposure index and FEV1 (r = -0.53, p = 0.004), FVC (r = -0.5, p = 0.008).

## Discussion

This cross-sectional study assessed the effect of VOC exposure on the respiratory symptoms and pulmonary functions of construction painters and paint industry workers working in unorganized sectors in and around Chennai. The concentrations of these VOCs in paints were found to be above the ACGIH TLVs among paint manufacturing workers and below the ACGIH TLV limits among construction painters. However, the concentrations of toluene, ethylbenzene, and xylene were shown to be greater in paint scraping and solvent brushing activities, exceeding the TLVs among construction painters.

Respiratory symptoms among paint industry workers

Respiratory symptoms were reported by 52% of the study participants. Inhaling VOCs dissolves pulmonary surfactants and changes their biphasic behavior, thereby increasing alveolar surface tension and decreasing lung compliance leading to dyspnea and chest tightness [14,15]. Exposure to these compounds may cause atypical inflammatory changes in the pulmonary vasculature, affecting small blood vessels and increasing collagen deposition, which increases mucous secretion and decreases ciliary mobility, causing coughing and sputum production [16,17].

Additionally, this study revealed that the prevalence of these respiratory symptoms was greater among construction painters in comparison to workers in the paint manufacturing industry. A plausible reason is that construction painters are exposed to elevated levels of respirable dust particles during paint scraping, in addition to potentially hazardous VOC exposure from old building paints during paint striping [4,18]. Nearly 82% of construction painters interviewed in the present study indicated that they were engaged in paint stripping and striping before painting, and this might be associated with a greater prevalence of respiratory symptoms. Similar findings have been reported by house painters [19], who stated that environmental dust or vapors pose significant challenges for workers. Besides, a longitudinal study conducted [20] among Swedish painters revealed that water-based paints were perceived to be less irritating and cause fewer respiratory symptoms.

Pulmonary functions among paint industry workers

While assessing the pulmonary functions using Wright’s peak flow meter it was found that 22% of the paint industry workers from unorganized sectors had reduced pulmonary function (PEFR<400 L/min). Further, it was found that there was a significant mild negative correlation between work experience and PEFR levels. Few animal studies have shown that since VOCs are lipophilic, they may penetrate the cell membrane and trigger oxidative stress-induced inflammatory changes in the lung parenchyma, leading to airway obstruction [16].

Spirometric assessment among a subset of randomly selected construction painters who were predominantly (reported >90% usage) exposed to solvent-based paints showed a significant reduction in all the PFT parameters like FEV1, FVC, FEV1 / FVC. Furthermore, there was a significant moderate negative correlation between FEV1, and FVC with both work experience and cumulative VOC exposure index. Several epidemiological studies have reported similar findings [10,21-23], however, few studies conducted among organized sectors like shipyard and automobile industries have shown no differences in the PFT parameters such as FEV1 and FVC among painters compared to that of the matched unexposed individual [24,25]. A few of the contributing factors that might reduce the pulmonary functions in these unorganized sectors are inadequate health awareness, lack of usage of PPE, and non-adherence to safety measures and protocols by the employees and the employers of these sectors [26]. Our study results also showed a significant reduction in FEV1, FVC, with advancement in age which was consistent with the findings of Metwally et al. who reported a significant negative correlation between lung functions with age and duration of exposure [27]. However, findings from other studies reported no significant correlation between age and pulmonary functions [28].

Although 73% of the study participants reported awareness about PPE usage, only six percent reported effective usage of PPEs in the form of cloth masks. A few of the common reasons stated by the painters for improper usage of PPEs were excessive heat, profuse sweating, uncomfortable to wear, and not being able to breathe properly, This is true, especially among paint industry workers from tropical countries like India where excessive exposure to heat may cause hindrance for the usage of PPEs.

One of the study's key strengths is an assessment of the concentration of VOCs using personal exposure monitors among the paint industry workers. In addition, the assessment of pulmonary functions among painters and small-scale paint manufacturing workers was done in unorganized sectors where recruitment and assessment are challenging.

A few of the logistic issues we faced while doing this research was acquiring permission from the owners of these unorganized sectors, who generally do not follow any safety rules or protocols. Further few eligible research participants, declined to participate due to the fear of unemployment if found to be unhealthy and unfit this might have introduced healthy worker bias and may have influenced the results. Additionally, noncompliance with protocols, such as abstaining from alcohol before the health examination, impeded the conduct of the study. Furthermore, the assessment of the temporal relationship was unattainable as a result of the study design. However, this study paves the way for future longitudinal research to identify true causality, particularly among workers in unorganized sectors where there are no particular government rules or protocols to monitor safety measures.

## Conclusions

Paint industry workers in unorganized sectors are exposed to higher BETX and dichloromethane levels, particularly among paint manufacturing workers. A higher prevalence of respiratory symptoms and diminished pulmonary functions was found in these workers. Increased work experience, higher cumulative VOC exposure index, and age affect pulmonary function. While the majority of painters in these sectors understand the importance of personal protective equipment (PPE), only a few use it. To reduce negative consequences, it is critical to enhance employee and employer understanding of occupational safety and services, as well as health and safety rules. In addition, the government should assist in drafting safety laws and perform frequent safety evaluations for unorganized sector workers to reduce morbidity
